# Mitochondrial DNA variants of Podolian cattle breeds testify for a dual maternal origin

**DOI:** 10.1371/journal.pone.0192567

**Published:** 2018-02-20

**Authors:** Piera Di Lorenzo, Hovirag Lancioni, Simone Ceccobelli, Licia Colli, Irene Cardinali, Taki Karsli, Marco Rosario Capodiferro, Emine Sahin, Luca Ferretti, Paolo Ajmone Marsan, Francesca Maria Sarti, Emiliano Lasagna, Francesco Panella, Alessandro Achilli

**Affiliations:** 1 Dipartimento di Scienze Agrarie, Alimentari e Ambientali, Università degli Studi di Perugia, Perugia, Italy; 2 Dipartimento di Chimica, Biologia e Biotecnologie, Università degli Studi di Perugia, Perugia, Italy; 3 Institute of Zootechnics, Università Cattolica del S. Cuore, Piacenza, Italy; 4 Biodiversity and Ancient DNA Research Center–BioDNA, Università Cattolica del S. Cuore, Piacenza, Italy; 5 Department of Animal Science, Faculty of Agriculture, University of Akdeniz, Antalya, Turkey; 6 Dipartimento di Biologia e Biotecnologie “L. Spallanzani”, Università di Pavia, Pavia, Italy; 7 Korkuteli Vocational School, University of Akdeniz, Antalya, Turkey; University of Florence, ITALY

## Abstract

**Background:**

Over the past 15 years, 300 out of 6000 breeds of all farm animal species identified by the Food and Agriculture Organization of the United Nations (FAO) have gone extinct. Among cattle, many Podolian breeds are seriously endangered in various European areas. Podolian cattle include a group of very ancient European breeds, phenotypically close to the aurochs ancestors (*Bos primigenius*). The aim of the present study was to assess the genetic diversity of Podolian breeds and to reconstruct their origin.

**Methodology:**

The mitochondrial DNA (mtDNA) control-regions of 18 Podolian breeds have been phylogenetically assessed. Nine non-Podolian breeds have been also included for comparison.

**Conclusion:**

The overall analysis clearly highlights some peculiarities in the mtDNA gene pool of some Podolian breeds. In particular, a principal component analysis point to a genetic proximity between five breeds (*Chianina*, *Marchigiana*, *Maremmana*, *Podolica Italiana* and *Romagnola*) reared in Central Italy and the Turkish Grey. We here propose the suggestive hypothesis of a dual ancestral contribution to the present gene pool of Podolian breeds, one deriving from Eastern European cattle; the other arising from the arrival of Middle Eastern cattle into Central Italy through a different route, perhaps by sea, ferried by Etruscan boats. The historical migration of Podolian cattle from North Eastern Europe towards Italy has not cancelled the mtDNA footprints of this previous ancient migration.

## Introduction

Over the past 15 years, 300 out of 6000 livestock breeds identified by Food and Agriculture Organization of the United Nations (FAO) have gone extinct [1]. Risk factors for farm animal breeds are mainly: i) a reduction of genetic variability due to strict selection processes; ii) a strong economic pressure focused on specific traits, such as milk production, which leads to the replacement of local less productive breeds with highly productive industrial breeds; iii) an unrestricted and indiscriminate cross-breeding, especially in developing countries [2]. *Bos taurus* is one of the most economically important livestock species [3]. Both in historic and current societies it has fulfilled agricultural, economic, cultural, and even religious key roles, often paralleling human evolution [4]. Among cattle, many Podolian breeds are seriously endangered in various European countries [5]; [6]; [7]. Podolian cattle include a group of very ancient European breeds, with a grey coat colour and long horns, phenotypically close to the aurochs (*Bos primigenius*). According to many traditional notes the name Podolian refers to a common ancestral origin in Podolia (the modern western Ukraine). However place of origin and timing of spread out of the source area are both debated. Alternative hypotheses have been proposed: Podolian cattle might have spread from the eastern steppe southward into Anatolia and westward into the Balkans and Italy in historical times (3^rd^-5^th^ century AD) along with East-European Barbarian people [8]; other authors suggest a more ancient migration (~3 kya BP) from the Near East to Central Italy through the Mediterranean Sea [9], together with a possible contribution from local wild aurochs through a secondary local domestication/introgression events[10]; [11].

Nowadays, some phenotypic distinctions stand out among Podolian cattle [12]. The noble aurochs-shaped ancient breeds with long horns (such as Hungarian Grey, Katerini, Podolsko, Slavonian Syrmian and Maremmana) are considered as the only true Podolian breeds by some scholars. However, some local breeds (i.e. Podolica Italiana, Ukrainian Grey, Turkish Grey and other Balkan breeds) do not necessarily show the long horns, but maintained some distinctive Podolian traits such as a red coat in calves and light grey in adults [13]. In general, a commercial trait shared by all Podolian cattle is that they are more suitable for beef production rather than for dairy. Because of that some improved beef breeds (Chianina, Marchigiana, Romagnola and Piemontese) are also considered within the Podolian group, although the inclusion of Chianina and Piemontese is still debated [14].

During the last decades, mitochondrial DNA (mtDNA) has been widely used as a molecular tool to investigate genetic origin, history and diversity of livestock species [15] [16] [17] [18] [19] [20]. Following this trend, the aim of the present study is to re-assess the mtDNA diversity of the major Podolian cattle breeds (ten of which classified as endangered or critical by FAO) to obtain additional information on their ancestral origin and ancient dispersal routes.

## Results

**Fig 1:**
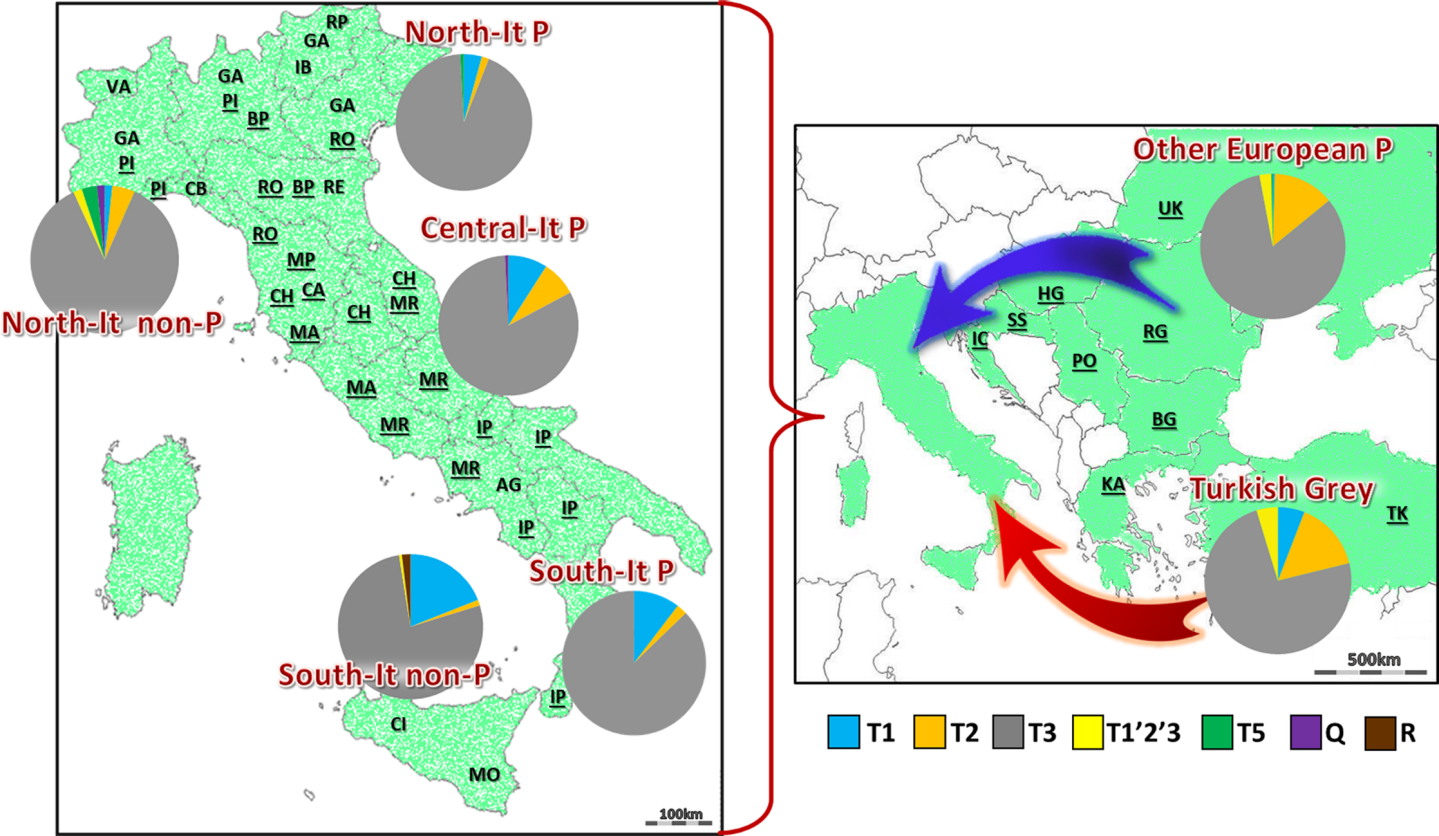
Prevalent locations and frequency distributions of mitochondrial haplogroups in 18 Podolian (P, underlined) and 9 non-Podolian (non-P) breeds analyzed in this study. Breed codes as in Table 1 (see also Table 2 and S3 Table for details). Note that RP and IB are also widespread in Italy. Maps (www.histgeo.ac-aix-marseille.fr/ancien_site/carto/) were used for illustrative purposes only and largely modified by the authors.

We have analysed the mitochondrial DNA of 18 Podolian cattle breeds (Fig 1, Table 1, S1–S3 Tables).

**Table 1:** Estimates of genetic diversity^a^ on the 27 breeds analyzed in this work.

Breed	Country	Podolian/non-Podolian	ID name	N	π	Hd	N	π	Hd
				Range:	16042-	16262	Range:	15823-	215
Piemontese	Northern Italy	Podolian	PI	72	0.008	0.833	72	0.005	0.970
Bianca di Val Padana	Northern Italy	Podolian	BP	45	0.006	0.630	45	0.003	0.834
Romagnola	Central Italy	Podolian	RO	225	0.014	0.890	225	0.007	0.965
Mucco Pisano	Central Italy	Podolian	MP	33	0.006	0.788	33	0.004	0.814
Calvana	Central Italy	Podolian	CA	35	0.008	0.662	26	0.004	0.889
Chianina	Central Italy	Podolian	CH	369	0.013	0.895	338	0.006	0.973
Maremmana	Central Italy	Podolian	MA	75	0.013	0.857	62	0.006	0.973
Marchigiana	Central Italy	Podolian	MR	146	0.013	0.903	146	0.006	0.967
Italian Podolian	Southern Italy	Podolian	IP	125	0.008	0.780	91	0.005	0.872
Ukrainian Grey	Ukraine	Podolian	UK	32	0.010	0.856	1		
Romanian Grey	Romania	Podolian	RG	17	0.014	0.890	-		
Hungarian Grey	Hungary	Podolian	HG	93	0.010	0.856	1		
Slavonian Syrmian Pod.	Croatia	Podolian	SS	9	0.004	0.583	-		
Istrian Cattle	Croatia	Podolian	IC	17	0.010	0.794	-		
Podolsko	Serbia	Podolian	PO	11	0.005	0.709	-		
Bulgarian Grey	Bulgaria	Podolian	BG	36	0.014	0.838	30	0.005	0.779
Katerini	Greece	Podolian	KA	12	0.019	0.879	-		
Turkish Grey	Turkey	Podolian	TK	85	0.012	0.922	70	0.006	0.971
Valdostana	Northern Italy	non-Podolian	VA	54	0.011	0.934	54	0.006	0.941
Grey Alpine	Northern Italy	non-Podolian	GA	45	0.012	0.853	45	0.006	0.979
Italian Brown	Northern Italy	non-Podolian	IB	34	0.010	0.852	34	0.005	0.929
Italian Red Pied	Northern Italy	non-Podolian	RP	136	0.010	0.896	125	0.006	0.982
Cabannina	Northern Italy	non-Podolian	CB	55	0.011	0.882	43	0.005	0.928
Reggiana	Northern Italy	non-Podolian	RE	38	0.006	0.713	38	0.003	0.845
Agerolese	Southern Italy	non-Podolian	AG	36	0.014	0.913	36	0.006	0.956
Cinisara	Southern Italy	non-Podolian	CI	81	0.014	0.881	69	0.007	0.966
Modicana	Southern Italy	non-Podolian	MO	41	0.010	0.763	33	0.005	0.864
		**All Podolian**		**1437**	**0.010**	**0.837**	**1140**	**0.006**	**0.980**
		**Non-Podolian**		**520**	**0.011**	**0.879**	**477**	**0.006**	**0.963**
		**All samples**		**1957**	**0.010**	**0.845**	**1617**	**0.006**	**0.977**

^a^ N = number of sequences

π = nucleotide diversity, Hd = haplotype diversity.

The molecular analysis of 221 base pairs of the control region (from np 16042 to np 16262) on the entire dataset of 1,957 samples revealed a total of 247 distinct haplotypes (from four to 70 haplotypes per breed) and 91 polymorphic sites (S), all represented by single nucleotide polymorphisms (SNPs). The average nucleotide diversity (π) was comparable between Podolian and non-Podolian breeds (~0.010–0.011; Table 1), while haplotype diversity was significantly lower (*P-value* < 0.01) in Podolian (Hd = 0.837 ± 0.010) than in non-Podolian breeds (Hd = 0.879 ± 0.013). Both indices varied largely across breeds as already seen in previous mtDNA studies [11]; [21]; [22]; [23]. Among all Podolian, the highest Hd values (≥0.90) were identified in Chianina, Marchigiana and Turkish Grey, while the lowest values of Hd (<0.70), as well as of nucleotide diversity (≤0.008), were scored in Bianca di Val Padana, Calvana, and Slavonian Syrmian Podolian. As for 1,617 sequences, we were able to extend the analysis to a longer control-region fragment encompassing 731 bps (Table 1). The results largely confirmed the same trend, with the only notable exception of Piemontese, Romagnola and Maremmana showing a higher Hd (>0.960) on this extended fragment. It is also interesting that the highest Hd values were identified in Chianina, and Maremmana, which showed values (>0.970) comparable to the Turkish Grey (0.971).

All control-region haplotypes have been classified in haplogroups and sub-haplogroups through an accurate analysis of mutational motifs (Table 2 and S3 Table), according to previously published classification criteria [11]; [24]; [25]; [26]. Haplogroup T3 was the most common (83%) in all breeds, with the highest value in MP and PO (both 100%), followed by PI (96%) (acronyms are listed in Table 1). The second and third most common haplogroups (both 7%) were T1 and T2, which were missing in MP, PO and SS. The frequency of T2 is lower in non-Podolian (3.85%) than in Podolian breeds (8.35%) with extraordinary high peaks in three breeds, KA (42%), RG (24%) and BG (22%), followed by UK (16%) and MA and TK (both 15%). T1 haplogroup was predominantly found among breeds from Central and Southern Italy, both in Podolian (10%) and non-Podolian (19%) groups (Fig 1). Haplogroup T5 was found exclusively in non-Podolian breeds, and was restricted to IB, RP and VA except for one sequence found in SS and one in PI. Finally, haplogroups Q and R showed very low incidences restricted to Italian non-Podolian (Q = 1.15%, and R = 0.58%) and Podolian (Q = 0.77%, and R = 0.49%) breeds. Overall, the haplogroup distribution differed significantly between the Podolian and non-Podolian groups of breeds included in the current analysis (Table 2; *chi-square P-value* < 0.001) with the highest contribution given by the T2 haplogroup. This result was also verified by considering haplogroup frequencies based on different haplotypes (*chi-square P-value* < 0.001) in order to mitigate the effect of inbreeding and recent founder effects.

**Table 2:** Sources and haplogroup affiliation for the Podolian and non-Podolian mtDNA sequences. Haplogroup frequencies (%) are in parentheses.

Code	Group/Breed	T1	T2	T3	T1'2'3	T5	Q	Q1	Q2	R	R1	R2	TOTAL
	**Podolian**	**103(7.17)**	**120(8.35)**	**1184(82.39)**	**10(0.70)**	**2(0.14)**	**1(0.07)**	**5(0.35)**	**5(0.35)**	**0(0.00)**	**5(0.35)**	**2(0.14)**	**1437**
**BP**	Bianca di Val Padana	5(11.11)	**-**	40(88.89)	**-**	**-**	**-**	**-**	**-**	**-**	**-**	**-**	45
**PI**	Piemontese	**-**	2(2.78)	69(95.83)	**-**	1(1.39)	**-**	**-**	**-**	**-**	**-**	**-**	72
**CA**	Calvana	5(14.29)	**-**	30(85.71)	**-**	**-**	**-**	**-**	**-**	**-**	**-**	**-**	35
**CH**	Chianina	35(9.49)	36(9.76)	292(79.13)	**-**	**-**	1(0.27)	2(0.54)	3(0.81)	**-**	**-**	**-**	369
**MR**	Marchigiana	18(12.33)	5(3.42)	122(83.56)	**-**	**-**	**-**	**-**	**-**	**-**	**-**	1(0.68)	146
**MA**	Maremmana	9(9.33)	11(14.67)	55(73.33)	**-**	**-**	**-**	**-**	**-**	**-**	**-**	**-**	75
**MP**	Mucco Pisano	**-**	**-**	33(100.00)	**-**	**-**	**-**	**-**	**-**	**-**	**-**	**-**	33
**RO**	Romagnola	12(5.33)	19(8.44)	183(81.33)	**-**	**-**	**-**	3(1.33)	2(0.89)	**-**	5(2.22)	1(0.44)	225
**IP**	Podolica Italiana	13(10.40)	3(2.40)	109(87.20)	**-**	**-**	**-**	**-**	**-**	**-**	**-**	**-**	125
**HG**	Hungarian Grey		8(8.60)	82(88.17)	3(3.23)	-	-	-	-	**-**	**-**	**-**	93
**UK**	Ukrainian Grey	-	5(15.63)	25(78.13)	2(6.25)	-	-	-	-	**-**	**-**	**-**	32
**BG**	Bulgarian Grey	1(2.78)	8(22.22)	27(75.00)	-	-	-	-	-	**-**	**-**	**-**	36
**IC**	Istrian Cattle	-	1(5.88)	15(88.24)	1(5.88)	-	-	-	-	**-**	**-**	**-**	17
**PO**	Podolsko	-	-	11(100.0)	-	-	-	-	-	**-**	**-**	**-**	11
**RG**	Romanian Grey	-	4(23.53)	13(76.47)	-	-	-	-	-	**-**	**-**	**-**	17
**SS**	Slavonian Syrmian Pod.	-	-	8(88.89)	-	1(11.11)	-	-	-	**-**	**-**	**-**	9
**KA**	Katerini	**-**	5(41.67)	7(58.33)	**-**	**-**	**-**	**-**	**-**	**-**	**-**	**-**	12
**TK**	Turkish Grey	5(5.88)	13(15.29)	63(74.12)	4(4.71)	-	-	-	-	-	-	-	85
	**Non-Podolian**	**36(6.92)**	**20(3.85)**	**435(83.65)**	**8(1.54)**	**12(2.31)**	**3(0.57)**	**3(0.58)**	**-**	**2(0.38)**	**1(0.19)**	**-**	**520**
**AG**	Agerolese	7(19.44)	**-**	27(75.00)	1(2.78)	**-**	**-**	**-**	**-**	**-**	1(2.78)	**-**	36
**CB**	Cabannina	**-**	5(9.09)	40(72.73)	7(12.73)	**-**	3(5.45)	**-**	**-**	**-**	**-**	**-**	55
**CI**	Cinisara	17(20.99)	2(2.47)	60(74.07)	**-**	**-**	**-**	**-**	**-**	2(2.47)	**-**	**-**	81
**GA**	Grigio Alpina	**-**	2(4.44)	41(91.11)	**-**	**-**	**-**	2(4.44)	**-**	**-**	**-**	**-**	45
**IB**	Bruna Italiana	2(5.88)	**-**	29(85.29)	**-**	3(8.82)	**-**	**-**	**-**	**-**	**-**	**-**	34
**RP**	Pezzata Rossa Italiana	2(1.47)	10(7.35)	122(89.71)	**-**	1(0.74)	**-**	1(0.74)	**-**	**-**	**-**	**-**	136
**MO**	Modicana	6(14.63)	**-**	35(85.37)	**-**	**-**	**-**	**-**	**-**	**-**	**-**	**-**	41
**RE**	Reggiana	2(5.26)	**-**	36(94.74)	**-**	**-**	**-**	**-**	**-**	**-**	**-**	**-**	38
**VA**	Valdostana	**-**	1(1.85)	45(83.33)	**-**	8(14.81)	**-**	**-**	**-**	**-**	**-**	**-**	54
	**Total**	**139(7.10)**	**140(7.15)**	**1619(82.73)**	**18(0.92)**	**14(0.712**	**4(0.20)**	**8(0.41)**	**5(0.26)**	**2(0.10)**	**6(0.31)**	**2(0.10)**	**1957**

**Fig 2:**
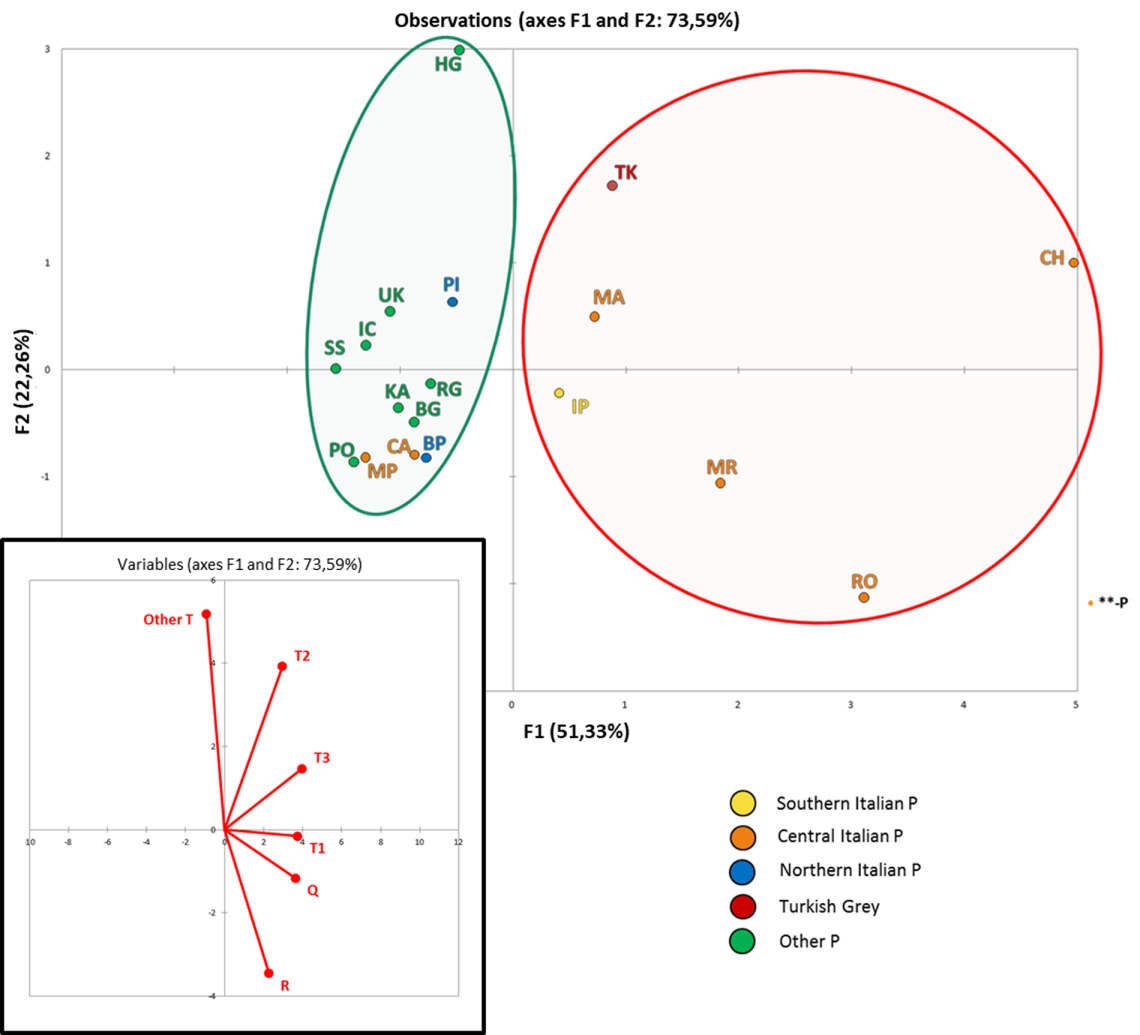
Principal Component Analysis (PCA) of all Podolian breeds. Below is the plot of the contribution of each haplogroup to the first and second PC (projections of the axes of the original variables).

Thus, we performed a principal component analysis (PCA) to graphically display the different haplogroup distributions among the Podolian breeds. In order to consider as many populations as possible, the dataset based on the short fragment was included. After variables reduction to PCs, the coordinates of the observations for the 18 breeds were displayed in a two-dimensional plot representing the European Podolian genetic landscape (Fig 2). PC1 clearly separated the Turkish Grey and the five most important Central and Southern Italian beef cattle breeds (CH, RO, MR, MA and IP; S1 Fig) from all the remaining populations, while PC2 contributed to separate the Hungarian and the Turkish Grey.

Because of the peculiar position of some Italian breeds, we used an analysis of molecular variance (AMOVA) to investigate fixation indices in three (artificially created) population groups, one including the Italian Podolian breeds, the other encompassing the European Podolian breeds and the Turkish Grey, and the third group covering the Italian non-Podolian breeds. Most of the variance (about 98%) observed in the Italian Podolian populations explained differences among samples within breeds, while less than 2% represented differences between breeds (Table 3), a value three times lower than in the other Podolian breeds.

**Table 3:** Analysis of molecular variance (AMOVA).

Group	Variation within breeds (%)	Variation among breeds (%)
**Italian Podolian breeds**	98.08	1.92
**Non-Italian Podolian breed**	93.85	6.15
**Non-Podolian breeds**	97.21	2.79

## Discussion

To date, only a limited number of studies have addressed the genetic composition of Podolian breeds. These investigations were generally limited to few breeds and focused on the nuclear genome [8]; [27]; [28]; [29]; [30]. Mitochondrial DNA data have been previously reported by Ivankovic et al. [6] and Ilie et al. [7] who analysed the control-region sequences of some Croatian and Romanian cattle breeds, respectively. The present study extended the analysis of the mitochondrial genetic variation to 18 Podolian breeds by evaluating their haplogroup distributions, which were eventually compared among them and to nine non-Podolian breeds. Genetic diversity considered in terms of number of haplotypes and nucleotide diversity revealed some peculiarities of several Podolian breeds. The low mtDNA diversities of Bianca di Val Padana, Calvana and Slavonian Syrmian Podolian could be due to a combination of factors, such as i) a sampling bias depending on the low consistence of current herds (S1 Table), ii) a bottleneck effect caused by the strong reduction in population size experienced by these breeds during the last decades, iii) genetic drift acting on small populations. On the contrary, the large diffusion of Piemontese, Marchigiana and Chianina cattle probably favoured the accumulation and maintenance of a high level of mtDNA variation, which is evident also in the Maremmana breed in spite of its lower consistency (S1 Table). In general, the most common European haplogroup T3 is predominant (83%) in our dataset. High frequencies of T1 in central and southern Italy might be due to the intensive migrations across the Mediterranean Sea, eased by the proximity to northern Africa, where T1 is prevalent [20]; [25]; [26]; [31] and to the Near East where T1 is also present [32, 33]. It is worth noting that the central-Italian Podolian breeds show higher frequencies of T2, while the presence of Q and R within the Podolian group is limited to three Italian breeds: Romagnola, Chianina and Marchigiana, the latter derived from crossbreeding between the first two in the early 20th century.

The significant differences between the haplogroup distributions of Podolian and non-Podolian and the low genetic differentiation among the Italian Podolian breeds (three times less than in other Podolian breeds or in the non-Podolian group) points to a common (and perhaps peculiar) origin. As a matter of fact, according to the first component of the PCA, five Italian beef cattle breeds formed a clearly separated group and were also closer to the Turkish Grey than to any other Podolian breeds. At first this peculiarity could be explained as the effect of a stronger beef-oriented selection carried out on these breeds compared to the other Podolian populations. However, another important Italian beef cattle, the Piemontese, is placed within the other Podolian cluster, which shares the common feature of a strong grey coat. Thus, an alternative explanation might assume a different ancestral origin for the two groups of Podolian breeds, as summarized in Fig 1: a first group, mostly consisting of breeds from East Europe and northern Italy that share a similar mitochondrial gene pool, may have originated from ancestors migrated through an inland route from Podolia across eastern Europe all the way into northern Italy, in accordance with the great wave of cattle migration occurred during the Barbarian invasions. Whereas, a second group, including the white Podolian cattle closely related to the Turkish Grey, may descend from ancestral bovines brought to Italy through a different and likely maritime route crossing the Mediterranean Sea. A previous study [34] suggested also a genetic link between the Turkish Grey and Bulgarian and Hungarian breeds, but our results do not support such hypothesis, highlight instead a stronger maternal relationship between the Turkish Grey and five central-southern Italian Podolian breeds. Those cattle are bred since the medieval time [29] in an area that largely overlaps with the ancient territory of Etruria. This finding further supports and extends another hypothesis, according to which at least part of the maternal ancestry of those breeds could be related to the Etruscan migration from Lydia, a region on the south-western coast of ancient Anatolia [9]; [35]; [36], [37]; [38]. It is worth noting that the five Podolian breeds are also the main Italian beef cattle together with the Piemontese, and that previous studies suggested a possible common genetic origin [39]. Our findings suggest that, in spite of a stronger beef-oriented selection, their mitochondrial gene pool still preserves genetic traces of a different maternal origin, confirming that the selection practices were mostly male-mediated and enforcing the importance of the mtDNA screening to reconstruct the ancestry and history of current breeds.

## Material and methods

### Ethics statement

All experimental procedures were reviewed and approved by the Animal Research Ethics Committee of the Universities of Perugia and Pavia in accordance with the European Union Directive 86/609.

### Samples

The entire dataset analyzed in this study encompasses 1,957 mtDNA control-region sequences including 1,321 from our previous studies [11]; [24]; [40], 428 retrieved from GenBank, and 208 additional samples specifically collected for this study (Table 1, S1–S3 Tables). Piemontese (also called Piedmontese) (PI, n = 72), Romagnola (RO, n = 225), Marchigiana (MR, n = 146), Chianina (CH, n = 369), Maremmana (MA, n = 75), Podolica Italiana (also known as Italian Podolian) (IP, n = 125), Mucco Pisano (MP, n = 33), Calvana (CA, n = 35), Bianca di Val Padana (BP, n = 45), Hungarian Grey (HG, n = 93), Bulgarian Grey (BG, n = 36), Istrian cattle (IC, n = 17), Katerini (KA, n = 12), Romanian Grey (RG, n = 17), Slavonian Syrmian Podolian (SS, n = 9), Turkish Grey (TK, n = 85), Ukrainian Grey (UK, n = 32), Podolsko (PO, n = 11). Moreover, nine unrelated non-Podolian breeds from Italy, are included as a control group: Valdostana (VA, n = 54), Bruna Italiana (also known as Italian Brown) (IB, n = 34), Grigio Alpina (also called Grey Alpine) (GA, n = 45), Pezzata Rossa Italiana (also known as Italian Red Pied) (RP, n = 136), Modicana (MO, n = 41), Reggiana (RE, n = 38), Agerolese (AG, n = 36), Cinisara (CI, n = 81), Cabannina (CB, n = 55).

### DNA extraction, amplification and sequencing

As for the 208 novel mtDNAs, blood samples were collected from the jugular vein of each animal in vacutainer tubes, containing EDTA as anticoagulant. These animals were chosen in different farms in order to avoid closely related individuals and gather a representative sample of the breeds. Whole blood was stored at -20°C until DNA extraction. DNA was isolated using the GenElute Blood Genomic DNA kit (Sigma Aldrich, St. Louis, MO, USA) and stored at -20°C until genotyping. PCR amplification of the control region was performed using forward and reverse primers (5’-CCTAAGACTCAAGGAAGAAACTGC-3’ and 3’-AACCTAGAGGGCATTCTCACTG-5’ respectively) specifically designed on the Bovine Reference Sequence (BRS; GenBank V00654). The 1138 bp PCR fragment encompassed the mtDNA control region from np 15718 to 517. Amplicons were first purified using exonuclease I and alkaline phosphatase (ExoSAP-IT® enzymatic system-USB Corporation, Cleveland, OH, USA), then sequenced with the primer 15757F (5’-CCCCAAAGCTGAAGTTCTAT-3’), as previously described [40]. A dataset of 1,321 sequences was already available in our laboratories. All data were recorded in GenBank with accession numbers MF474376-MF475904 (S3 Table) and compared to those retrieved from the database (S2 Table).

### Data analyses

Sequences were aligned to the Bovine Reference Sequence (BRS; V00654) using the software Sequencher^TM^ 5.0. For a total of 1,617 samples, we were able to analyse a 731-bps fragment trimmed from np 15823 to np 215, while only a short fragment (221 bps, from np 16042 to np 16262) was considered in order to include the widest possible number of samples (N = 1,957). Haplotypes were classified in haplogroups and sub-haplogroups according to previously identified mutational motifs [24]. Indices of molecular variation were calculated using the DNAsp 5.1 software [41], while an analysis of molecular variance was computed using AMOVA program implemented in the ARLEQUIN 3.01 package [42]. Finally, principal component analyses (PCA) were performed using Excel software implemented by XLSTAT, as described elsewhere [43, 44]. The PCA is a widely used dimension-reduction method that summarizes the variance of multivariate data in a smaller number of variables (the principal components, PCs), which are linear functions of the original variables, here expressed as haplogroup and sub-haplogroup frequencies, The rarest haplogroups were phylogenetically grouped and frequencies were calculated by considering only different haplotypes within the same breed.

## Supporting information

S1 Table List of Podolian breeds analyzed in this study. (XLSX)

S2 Table List of samples retrieved from GenBank. (XLSX)

S3 Table List of samples analysed in this study. (XLSX)

S1 Fig The five most important Italian beef cattle breeds from central and southern Italy discussed in this paper. (PDF)
