# Supplementary figures and images for: Mitochondrial DNA variants of Podolian cattle breeds testify for a dual maternal origin
Source: PLoS One. 2018 Feb 20;13(2):e0192567. doi: 10.1371/journal.pone.0192567 (PMC5819780; doi:10.1371/journal.pone.0192567)

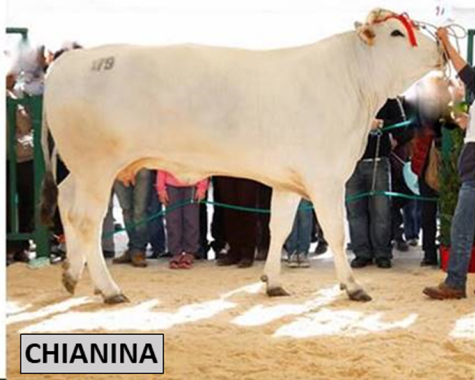

**CHIANINA**

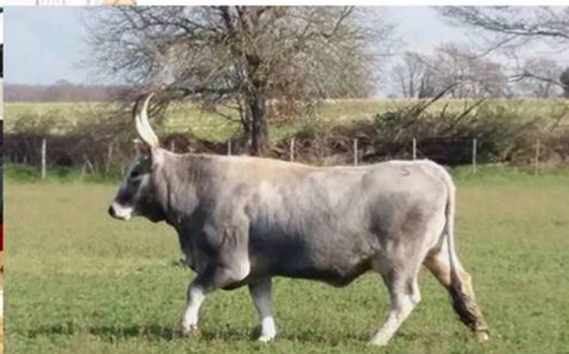

**MAREMMANA**

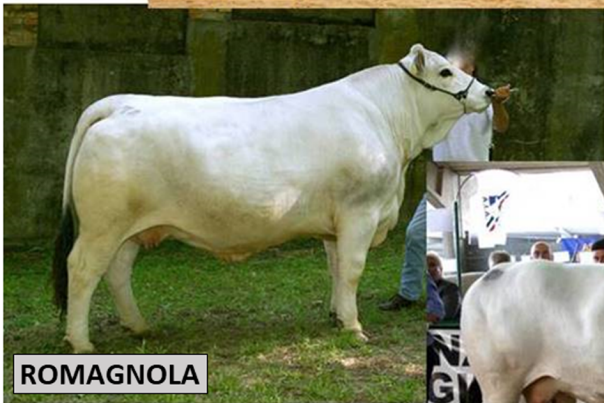

**ROMAGNOLA**

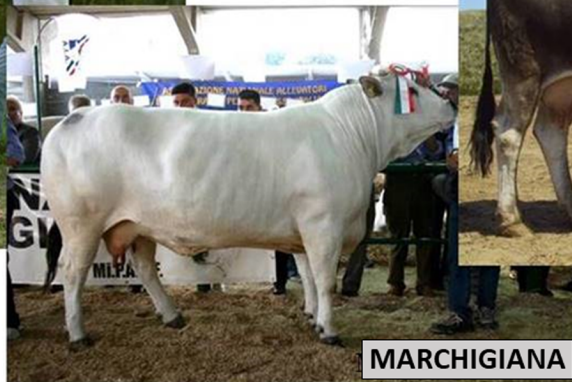

**MARCHIGIANA**

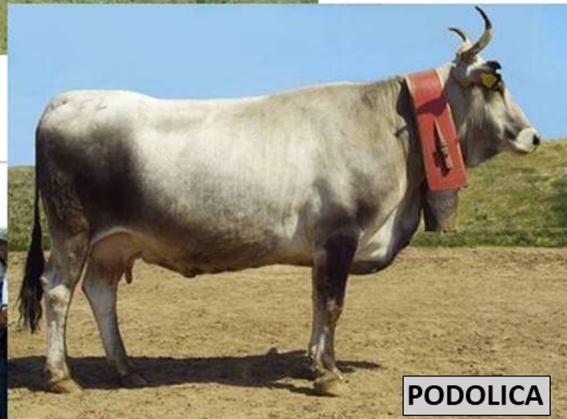

**PODOLICA**

Supplement: S1 Fig — (PDF) [file pone.0192567.s004.pdf]
